# Leber hereditary optic neuropathy presenting as bilateral visual loss and white matter disease

**DOI:** 10.6026/97320630019226

**Published:** 2023-03-31

**Authors:** Hussein Algahtani, Bader Shirah, Angham Abdulrhman Abdulkareem, Fehmida Bibi, Peter Natesan Pushparaj, Muhammad Imran Naseer

**Affiliations:** 1 Neurology Section, Department of Medicine, King Abdulaziz Medical City, Jeddah, Saudi Arabia; 2 King Abdullah International Medical Research Center, Jeddah, Saudi Arabia; 3 College of Medicine, King Saud bin Abdulaziz University for Health Sciences, Jeddah, Saudi Arabia; 4 Department of Neuroscience, King Faisal Specialist Hospital & Research Centre, Jeddah, Saudi Arabia; 5 Center of Excellence in Genomic Medicine Research, King Abdulaziz University, Jeddah, Saudi Arabia; 6 Faculty of Science, Department of Biochemistry, King Abdulaziz University, Jeddah 21589, Saudi Arabia; 7 Special Infectious Agents Unit, King Fahd Medical Research Centre, King Abdulaziz University, Jeddah 21589, Saudi Arabia; 8 Department of Medical Laboratory Technology, Faculty of Applied Medical Sciences, King Abdulaziz University, Jeddah, Saudi Arabia

**Keywords:** MT-CO3, Leber Hereditary Optic Neuropathy, Mitochondrial Inheritance, Acute Disseminated Encephalomyelitis, Saudi Arabia

## Abstract

Leber hereditary optic neuropathy (LHON) is a rare maternally inherited mitochondrial disorder that typically affects young male adults in their second and third decades of life. It usually manifests as painless, subacute, progressive, bilateral vision loss, with more than 90% of affected individuals losing their vision before age 50. Compared with other diseases that cause optic neuritis (multiple sclerosis or neuromyelitis optica spectrum disorders), LHON has worsening visual function in the first 6-12 months of disease progression, is predominantly male, the optic nerve is affected bilaterally from onset, there is no gadolinium enhancement on MRI, no response to disease-modifying therapy, and there is a family history of mutation in mitochondrial DNA. In this article, we describe an interesting and challenging case of LHON due to a homoplasmic variant in the MT -CO3 gene that was initially misdiagnosed as a monophasic demyelinating disorder (clinically isolated syndrome vs acute disseminated encephalomyelitis vs neuromyelitis optica spectrum disorders).

## Background:

Leber hereditary optic neuropathy (LHON) is a rare maternally inherited mitochondrial disorder that typically affects young male adults in the second and third decades of life with a prevalence of approximately 1-9/100000. It is usually manifested by painless, subacute, progressive, bilateral vision loss. More than 90% of affected individuals lose their vision before the age of 50. Due to its low penetrance (27% in men and 8% in women), only about 40% of patients know that their family members have symptoms of the disease [1]. Compared to other diseases that cause optic neuritis (multiple sclerosis or neuromyelitis optica spectrum disorders), LHON has worsening visual function in the first 6-12 months of disease progression, is predominantly male, the optic nerve is bilaterally affected from the onset, there is no gadolinium enhancement on MRI, no response to disease-modifying therapy, and there is a family history of a mutation in mitochondrial DNA [2]. In this article, we describe an interesting and challenging case of LHON due to a homoplasmic variant in the MT -CO3 gene that was initially misdiagnosed as a monophasic demyelinating disorder (clinically isolated syndrome vs. acute disseminated encephalomyelitis vs. neuromyelitis optica spectrum disorders). We also address visual prognosis and brain MRI changes in patients with LHON.

## Case Report:

**Figure 1:**
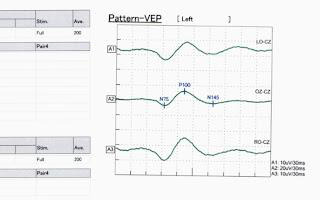
Visual evoked potential showing prolonged p-100 latencies in both eyes indicating involvement of the optic nerves.

**Figure 2:**
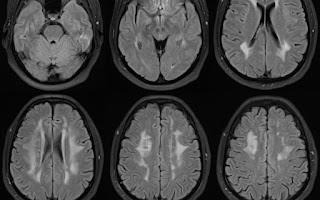
MRI of the brain showing large periventricular confluent white matter high signal intensity on FLAIR sequence

A 44-year-old man presented to the neurology clinic after experiencing sudden, painless bilateral vision loss for 3 days. He denied any history of ataxia, headache, seizures, numbness or tingling, weakness, or problems with sphincter control. He had no history of upper respiratory tract infections or immunizations. He is known to have diabetes and hypertension, which were diagnosed 5 years earlier, with no end-organ complications. He did not smoke or drink alcohol. There was no family history of similar disease or autoimmune disease. Clinically, the patient was conscious, oriented, and alert with normal vital signs, body mass index, and mini mental status examination. His vision was impaired in both eyes (finger counting). His pupil size was normal with normal response to light and accommodation. His fundus examination was normal with no papilledema, pallor, or atrophy. Assessment of the visual field was difficult. The rest of the cranial nerves, motor function, sensory function, cerebellum, and gait were normal, and there was no evidence of long tracts. The visual field (Humphrey 30-2) showed bilateral central scotoma. Visual evoked potential showed prolonged p-100 latencies in both eyes, indicating optic nerve involvement (Figure 1). Baseline hematologic and biochemical examinations, connective tissue examination, and vitamin deficiency assessment were all normal. MRI of the brain showed fairly symmetrical, diffuse, confluent, bilateral high signal intensity on T2 and FLAIR images in the periventricular, centrum semiovale, and subcortical white matter, including optic radiation (Figure 2). There was no diffusion restriction, pathologic enhancement, or mass effect. MRI of the orbits and entire spine was unremarkable. CT Angiography of the intracranial and extra cranial blood vessels was normal. CSF analysis revealed normal cells, glucose, and protein without oligoclonal bands. Based on the clinical presentation of a young, alcohol-free, and well-nourished man, acute monophasic demyelinating disease was suspected (clinically isolated syndrome vs. acute disseminated encephalomyelitis vs. neuromyelitis optica spectrum disorders). He was started on pulsatile steroid therapy followed by brief oral steroid therapy. Aquaporin-4 and anti- MOG serology were negative (repeated twice). The patient was reexamined in the clinic three months later, where his vision was normal, including visual acuity, fundus examination, visual field examination, and color vision. A repeat MRI of the brain showed persistence of the previously described white matter abnormalities. Because demyelinating disease could not explain the patient's clinical (simultaneous bilateral visual loss) and radiologic findings (unusual and atypical location of demyelinating plaques), genetic testing with targeted sequencing was performed in which the entire coding region of the genes MT -ATP6, MT -CO1, MT -CO3, MTCYB, MT -ND1, MT -ND2, MT -ND4, MTND4L, MT -ND5, and MT -ND6 was amplified and sequenced (Next Generation Sequencing of Leber optic atrophy panel). A variant in the MT -CO3 gene m.14790A > G p.CYTB: (Asn15Ser) was detected in a homoplasmic state (99.78% of 4861 NGS reads). The corresponding nucleotide and amino acid are highly conserved. Clinical evaluation of the patient, including visual assessment performed five years after diagnosis, still showed normal visual pathway functions and similar neuroimaging findings. Our final diagnosis in this patient five years after presentation was LHON. The patient was followed regularly and underwent annual MRI examinations of the brain, orbit, and entire spine to rule out the possibility of remote multiple sclerosis.

## Discussion:

The pathologic data on LHON are largely limited postmortem studies performed in elderly patients in whom visual loss had occurred several decades ago. The neuropathology in LHON is generally confined to the eye, particularly the retinal ganglion cell layer, leaving out the photoreceptors and retinal pigment epithelium. There is marked degeneration of the cell bodies and axons with associated demyelination leading to optic nerve atrophy extending to the lateral geniculate bodies. Although rare, pathologic changes can occur in the central nervous system, affecting the cerebral hemispheres, cerebellum, brainstem, and spinal cord. These changes range from extensive demyelination to cystic tissue destruction and necrosis [3]. No pathologic specimen was obtained in our patient. However, neuroimaging revealed extensive symmetrical demyelinated areas in the white matter with somewhat more localization in the frontal lobes compared with the other lobes. Previous pathologic studies suggest an autoimmune cause for these changes, mediated predominantly by T cells and activated macrophages/microglia [4]. Our patient's response to steroids with complete recovery of visual loss may support this hypothesis. Further pathologic studies on an international level with more autopsies and tissue pathology are recommended.

Neuroimaging (CT and MRI scans) are usually normal in LHON. MRI of the brain is useful to rule out other neurologic mimickers, especially multiple sclerosis. Abnormal radiological signs such as high white matter signal intensities can be observed in LHON, in patients with other mitochondrial gene defects, and in cases of LHON associated with other defects of the same gene [5]. These changes are sometimes difficult to distinguish from those seen in demyelinating diseases such as multiple sclerosis. Several clues can be used to distinguish between these two diseases, including the clinical course of the disease, family history of a genetic disorder, spinal cord imaging, cerebrospinal fluid analysis, and genetic testing [6]. In a study by Matthews et al [7], a blinded review of MRIs of the brain showed that differentiation based on imaging is difficult because the radiological appearance is strongly influenced by women. In our case, the initial suspected diagnosis was demyelinating disease, and the patient was treated as such. However, because of the lack of spatial and temporal spread, normal imaging of the spinal cord and optic nerve, bilateral painless visual loss, and atypical white matter changes on brain MRI, genetic testing was performed to rule out mitochondrial disease.

LHON can be misdiagnosed as a demyelinating disease. Several studies have shown that the risk of a patient developing LHON and multiple sclerosis is about fifty times higher than chance. This is because autoimmunity plays an important role in the development of mitochondrial disorders [8]. Neurologists should consider a genetic mitochondrial disorder in a patient diagnosed with multiple sclerosis, especially if atypical features are present (e.g., severe sequential optic neuritis episodes or progressive visual loss despite appropriate treatment). We believe that these two conditions are separate disorders and that this association is unlikely or represents a chance coincidence. Mitochondrial disease could explain the poor outcome and lack of response to disease-modifying therapy in these rare cases of what appears to be an autoimmune demyelinating disease of the central nervous system.

LHON is a serious cause of vision loss in early adulthood. It is usually manifested by acute or subacute progressive deterioration of visual acuity, and within a year of disease onset, patients exhibit pale optic discs and degeneration of retinal ganglion cells and their axons [9]. However, for unknown reasons, some patients may experience partial recovery of central vision in one or both eyes. This spontaneous visual recovery is a well-described phenomenon in LHON, usually occurring within the first year after visual loss. However, recovery may occur decades later. Certain mutation types are more likely to recover partially than others. Other prognostic factors include an age at onset of less than 20 years, a subacute time course, and large optic discs [10].

## Conclusion:

Demyelinating diseases can well mimic various neurologic diseases, including LHON. This can result in delayed diagnosis and unnecessary and potentially harmful medications, including disease-modifying therapies. Multidisciplinary care by a neurologist, neurologist, and ophthalmologist is strongly recommended. Return to baseline education, including history, physical examination, and differentiation of clinical features, supported by paraclinical investigations such as neuroimaging, is warranted, and a definitive diagnosis can be made based on genetic testing. Neuroradiologists should be aware of the possible MRI features of LHON.
